# The Challenges of Establishing Healthcare Services in Low- and Middle-Income Countries: The Case of Autism Spectrum Disorders (ASD) in the Kurdistan Region of Iraq—Report from the Field

**DOI:** 10.3390/brainsci12111433

**Published:** 2022-10-25

**Authors:** Sayyed Ali Samadi

**Affiliations:** Institute of Nursing and Health Research, Ulster University, Belfast BT15 1ED, UK; s.samadi@ulster.ac.uk

**Keywords:** autism spectrum disorders, developmental disabilities, establishing health services, healthcare services, low- and middle-income countries, Kurdistan region of Iraq

## Abstract

The present report from the field tries to present challenges associated with establishing healthcare services for individuals with autism spectrum disorders (ASD) in a low- and middle-income area. The given case that has been highlighted is establishing daycare services focusing on rehabilitation and training services for individuals with ASD, and their caregivers and family members, in the Kurdistan Region of Iraq (KRI). Based on my experience, the associated challenges are divided into three primary levels: personal, professional, and organizational. The report highlights the importance of understanding the challenges associated with establishing healthcare services with the desire to put them under control. Plus, understanding the cultural aspects of the healthcare services for individuals with ASD experiences to address the issues at each level shared. It is concluded in the final part of the report that the key to establishing healthcare quality is to understand attitudes toward healthcare at the personal, professional, and organizational levels. This attitude determines the expectation from the services and defines assistance-seeking behaviors. Results offer insight for stakeholders in the healthcare field, allowing for a better understanding and improvement of availability and access to quality-driven healthcare services. A pre- and in-service training approach is practiced to address the associated challenges of establishing healthcare services at the personal level in KRI. A similar policy might be applicable in other LMICs, where there is a lack of professional and skillful healthcare service providers.

## 1. Introduction

Autism Spectrum Disorder (ASD), as it is defined by the Fifth Edition of the *Diagnostic and Statistical Manual of Mental Disorders (DSM 5)* published by the American Psychiatric Association (APA), is a neurodevelopmental disorder. The diagnosis is associated with symptoms that include “persistent deficits in social communication and social interaction across multiple contexts” and “restricted, repetitive patterns of behavior, interests, or activities” [1]. Centers for Disease Control and Prevention (CDC) is a reliable base that is trying to provide essential data on this diagnosis to help understand factors that increase children at risk for ASD and its possible causes, and also to develop resources that help identify children with ASD as early as possible to provide services at the early stages of the symptom emergence [2].

The term low- and middle-income country (LMIC) is definable from different aspects, but in reality it refers only to the economic condition of nations. Based on their gross national income per capita, countries worldwide are divided by the World Bank into four economic groups: high, upper-middle, lower-middle, and low-income countries. Least developed countries are also called by other names, such as landlocked developing countries [3] and small island developing states (SIDS) [4]. Countries on the other end of the spectrum are usually high-income countries (HIC) or developed countries.

According to Saraceno et al. [5], service development has been slow despite promising activities regarding improving community health in several countries in most LMICs. Establishing services for children and adolescents is an urgent need [6], especially for those with different development trajectories. Services in most countries classified as LMIC mainly shape a group that provides health and care support, which is defined as community health workers. Lewin et al. [7] describe this group as “Any health workers who perform functions related to healthcare provision; having no formal professional or paraprofessional certificate or degree in tertiary education but trained in some way in the context of the intervention”. The dearth of human resources in the general field of health and evidence of the efficacy of the services provided by this group made them a critical factor in the healthcare provision in LMICs [8]. They are considered the cornerstones and drivers of health care services and interventions [9]. Nevertheless, there are serious concerns about this group’s competencies and skillfulness regarding independent service provision, data handling and analysis, and the importance of data use for improving service delivery in their area.

It is estimated that about 1–2% of children have a form of ASD globally [10]. The reported rate for different countries varies based on available studies. Although there are some reports about the prevalence of ASD in less-developed countries, [11] the exact prevalence rate in many LMICs is unknown, and most studies are performed in HICs. There is a shortage of reliable reports on ASD prevalence in Iraq. Hence, the World Population Review website predicted a rate of 89.40 in 10,000 [12]. Based on the available information from the Kurdistan Region of Iraq (KRI), the ASD rate is increasing in the area; according to the Center for Kurdistan Progress [13], a population exceeding three thousand children has been labeled as being under the autism spectrum. This number is a sizeable population with an absence of knowledge; the novelty of this review is that this group of children, their families, and essential required services and challenges associated with addressing these needs has rarely been reviewed. Observing the KRI’s health system for individuals with ASD provides many first-hand lessons about available ASD service reform barriers. Staying in the field for two years (from 2020 to 2022), as the healthcare system developer based on a formal invitation from a parental organization for children with ASD, provided valuable experience. Opportunities include consulting the high-ranked service providers in ministries of health and education and working with the professionals in both ministries.

The necessity of accessing the healthcare system is a known issue in LMICs. Individuals with disabilities, such as people with ASD in these countries, have a higher mortality rate due to a lack of essential healthcare support [14]. While many developmental disability advances have been made in support and services, they are primarily seen in affluent nations. These countries have seen reductions in disability mortality; however, in less developed countries, wealthy families can afford treatment expenses, and many lower socio-economic classes cannot afford the needed treatment or assistive devices. There is also a shortage of literature on individuals with ASD and their needs. The barriers to healthcare provision for individuals with ASD in KRI might be identified on at least three levels, personal, professional, or organizational, based on the present healthcare system in the area.

### 1.1. At the Personal Level

There are issues with two aspects of skills at this level. First non-technical personal skills of healthcare providers, problems with individualized healthcare providers’ organizational behavior, and lack of knowledge regarding teamwork in the field of healthcare provision in LMICs are labeled as “negative provider behaviors”. These skills are commonly defined as cognitive or social skills related to successfully executing a specific task or series of functions [15]. Some examples of these behaviors at the cognitive level are decision making, planning, and situation awareness, and at the social level are leadership, teamwork, and communication. The negative behaviors at this level are prioritizing personal benefits, being absent from work, soliciting informal payment for service delivery [16], and other similar activities.

The second personal level’s shortcoming is healthcare providing errors and engaging in disrespectful or abusive treatment or intervention, or treatment adopted from their anecdote and personal experience. These shortcomings impede quality caregiving, and contribute to poor service utilization, intervention and service provision outcomes. There are also questions about the suitability and relevance of the formal training and curriculum content, the suitability of bases through which training is delivered, and the impact of such training on service provision for individuals with ASD or other types of DDs.

### 1.2. At the Professional Level

Professionalism is defined as an output of the interaction between practitioner, client or service user, and context. The professional carries relatively stable beliefs, traits, and attitudes, but they must respond to the demands of the clients, personally as well as clinically. The organizational and physical environment must allow professionalism to flourish. Therefore, it is so localized in specific interactions that any fundamental approach to defining what is or is not professional will be problematic.

It is reported that in the health and developmental disabilities care field, service users and their families request specialist skills from the professionals: respect, clear communication, and behavior to reflect high standards of personal integrity. Health care professionals have focused on competency, which can be taught, developed, measured, and assessed [17]. Hence, findings indicate that considering professionalism as a holistic construct might be more beneficial [18]. From the holistic construct attitude, professionalism is any attempt to address issues beyond trainees’ educational situational behavior, and consider how to develop the working environments to ensure that standards are maintained post-registration and do not deteriorate in practice. It is also found that international management and organizational roles in determining professionalism were not identified. Increased awareness of their responsibility to support professional behavior may set new challenges for educators and regulators.

Professionalism is particularly critical in LMICs with a shortage of resources, where competence in their deployment is essential to meet the national and global targets for services and caregiving outcomes. In many LMIC settings, medical doctors are called upon to fill entire caregiving services establishments such as healthcare managing. The reason is their level of education, respected status in society, and clinical/technical expertise related to the particular medical services they can provide [19]. Nevertheless, medical doctors are rarely prepared for this role because of the lack of basic training, mentorship, and professional development in different fields of ASD. Clinicians in different caregiving managerial roles are often asked to simultaneously continue their clinical practice [20], which is rarely done in LMICs, particularly for medical doctors.

### 1.3. At the Organizational Level

As Bergström et al. [21] indicated, no existing tools are available for systematically recording or following up on the impact of organizational context’s aspects on implementing healthcare services in LMICS. It is reported that solid organizational commitment in the healthcare sector increases stakeholders’ participation levels, boosting the organizational culture, helping develop sustainable programs, and improving healthcare outcomes and the quality of provided services [22]. Hence, as a severe organizational shortcoming in the healthcare sector in LMICs, there is neglect in understanding and evaluating the healthcare providers’ importance and value for the position they are trained in. The essential element of this neglect is the absence of theoretical or conceptual models of understanding their role and an absence of data on healthcare providers’ perspectives about their duties. It was revealed that regardless of the shortage of professionals and the heavy load of healthcare providers in number, their value from the side of the authoritarian is underrated, although data imply that factors such as recruitment, training, and loss of organizational knowledge increase the overall cost for healthcare service providers in managing high employee turnover is substantial [23]. Hence, this critical factor is not fully understood by the authoritarians at an organizational level in LMICs.

The HICs are catching up on their delay in developmental disabilities standards they considered for the physical illness. Rosenbaum and Gorter [24] believe that the good news is that there are significant new ideas about health and childhood disability in the new century, helping us expand our thinking. The LMICs have to deal with similar issues to HICs; they also have additional factors to deal with: the persisting stigma of developmental disabilities and the focus of limited resources on physical illnesses, which results in developmental disabilities’ particular needs often being undetected or untreated. It may be particularly the case in populations with high social, emotional, and physical deprivation, factors known to increase rates of associated mental illness.

The main goal of the present report is to explain the fully-fledged program implemented in KRI, particularly for ASD, to understand its shortcomings at the main levels of personal, professional, and organizational, as a possible guideline to consider in establishing a healthcare system for children and adolescents with ASD and their caregivers. In addition, to expand the diagnosis and intervention monitoring systems already practiced in similar communities, and to develop acceptable standards for healthcare given to children and adolescents with ASD; to train health and administrative staff in the data collecting and management and analysis of health monitoring system data; to provide a basic model for dealing with other types of developmental disabilities; and to include private-sector daycare and healthcare systems in the health monitoring system, which plays a significant role in the KRI health system. A subsequent experience sharing for healthcare system providers in the region to improve their support and services.

## 2. The Kurdistan Region of Iraq Context

In KRI, similar to the entire country, there are reported shortages of formal and institutionalized structures focused on monitoring physical, mental, and developmental disabilities to make healthcare needs tangible [25]. There is also a lack of formal standards for different types of services and an ambiguity in professional healthcare providers’ duties and qualifications. The existing ambiguity is due to a lack of rehabilitation services such as speech and language therapy (SLT) and occupational therapy (OT). This results in limited services, inadequate knowledge of the population’s general health needs, insufficient understanding of their different demands, limited public health perspective and organization, and inability in prevention, rehabilitation, and intervention planning to meet the various domains of the current needs [26]. The present opinion article is based on nearly two years of working in the KRI to establish essential support and services for children with developmental disabilities, particularly children with autism spectrum disorders (ASD).

Kurdistan Region of Iraq (KRI) is an autonomous area that includes most of the Kurdish population in the northern part of Iraq, with borders with Iran, Syria, and Turkey. It is estimated that the population comprises 5.1 million in nearly 1 million households [27]. This area has accommodated groups of displaced Iraqis (1.2 million) and Syrian refugees (225,000) [28]. Available data predicted that 3% of individuals living in KRI present one disability—two thirds from a skeletal, disfiguring, or mobility impairment and less than one third an intellectual, mental and psychological impairment. The rate of disability prevalence is reported to be between 2.4% and 2.6%, indicating the presence of a lower rate of developmental disabilities among the internally displaced population. The possible reason for this difference is the younger structure of the substituted groups.

Thirty-five percent of the area’s population are younger than 15 years, with over half aged between 16 to 65 years. Forty-five percent of the population over six are illiterate [29].

Children with different types of disabilities, similar to children with ASD in this area, are usually diagnosed by physicians and medical staff such as psychiatrists or pediatricians, either at public medical facilities or privately. Although some governmental and private training and rehabilitation centers are available, there is a shortage of special education services for different age groups. Based on academic regulation, public and private schools accept children with high-functioning ASD or other milder forms of DDs. Some charities and private and governmental daycare centers provide essential services daily, and children with more severe conditions or disabilities are generally admitted to these centers, however, these services are not well established or available in rural areas, and are only affordable by affluent families who are residents of the large cities.

## 3. The Development of a Culturally Suitable Assessment and Intervention Service for Children with ASD in KRI

In KRI, the central problem in establishing health services and professional care provision is the lack of feasible planning for health care providers. The current services focus on short-term objectives such as basic daily caregiving activities, and there is a shortage of systematic plans for services for individuals with DDs in different stages of development. The existing centers primarily provide preschoolers with similar caregiving support without collecting reliable statistical data on the impacts of the services on the individuals during service provisions. Considering information systems and statistical data on an acceptable scale provides reliable factors for quality service establishment, and long-term planning with a focus on training and capacity building. These data and information are essential to support evidence-based decision making and manage available resources to allow a prompt response to future and emerging needs of individuals with DDs in different stages of development. Therefore, electronic data collecting systems are essential for long-term plans.

In February 2020, a center for children with ASD in Erbil tried to develop and update its services in light of a five-year action plan prepared by the author of this report after auditing the system. The center’s founder was an advocate and father of two children with ASD. The center had five years of previous service provision through a family business approach, without accessing professional training or supervision to help with meeting the required standards. The center aimed to provide assessments and interventions to most children and families, funded mainly by parental contributions.

The personnel were a multidisciplinary team of 35, comprised of psychologists, speech and language and occupational therapists (both from the neighboring countries), special educators (mainly local but with some educators from the neighboring countries), and support workers in the services section. The center advised adopting the bio-psycho-social development model within a social-ecological model embodied in the World Health Organization, the *International Classification of Functioning for Children and Youth*, with regards to clinical thinking and research and practice [30,31] based on the available evidence regarding its applicability with ASD service providers in similar cultural contexts [32]. The five-year action plan was prepared and activated in September 2020 (seven months after the first visit and auditing). It was planned to be finalized in September 2025. The key themes for this five-year action plan were: service redesign to improve ASD’s available home and community care, performance improvement of ASD service providers in the center, training and raising professionals, parents’ and caregivers’ engagement in the service provision, community awareness on ASDs, particular service to improve informational needs and to boost communication for individuals and families with ASD, and effective engagement and partnership working inside and outside the organization. The aim was to reduce the reported stigma experienced by caregivers of individuals with DDs, particularly those with ASD in LMICs [33], and to design culturally appropriate interventions to improve awareness about developmental disorders and ASD, decrease stigma, recover access to proper professional and general training, and strengthen caregiver-based services and support programs. The following bases were considered to attain the aims of the action plan possible:

1. Provide various opportunities for stakeholders to discuss different aspects of developmental disabilities and share other sectors’ knowledge and expertise in the area. This aim was attained by developing a board of managers for the center consisting of five stakeholders, including a representative from the parents;
2. Bridging the gap between ASD and other DDs research, policy, and practice and producing reliable data. Since a lack of data or relying on inaccurate data might create poor models for service provision, most children with disabilities live in LMICs. Still, there are unavailable or inaccurate data about them, and no amount of sophisticated modeling can compensate for poor primary data sources [34];
3. Exploring the available potential of sponsoring studies and research in developmental disabilities. Contacts with the ministry of health, education, and different public and private universities were engaged to achieve this aim;
4. Providing a supported network of developmental disabilities research, policy, and practice within the area and nearby countries. Without a rehabilitation and non-medical intervention background in the area, the main endeavor was to establish contacts with neighboring countries with more experience in this field.

To attain the three first themes in the first and second years, the following activities were performed: Preparation of the organization’s constitutions consisting of aims and objectives and organizational charts and different child- and family-centered protocols to assess and diagnose, and children’s placements in early intervention, daycare programs or clinical services. Consultation to guide the further development of the organization was done with international professionals in the field of ASD. The main aim was to develop a culturally adapted model for service provision for ASD (that might act as a model for other DDs) in the region.

Based on the previous experience in Iran and the Iranian Kurdish areas and adopting standard cultural features of the Kurdish culture in both countries, early intervention involved a one-day home visit during the week of four days of 4 h of training, based on the Denver Model established for children under five years old. Over five-year-old children’s daycare services developed to five days in the center in a four-hour program.

The entire training and rehabilitation staff revised 12 days of the workshop format training on Applied Behavior Analysis (ABA), and structured teaching with an emphasis on the TEACCH approach (Treatment and Education of Autistic and Related Communication Handicapped Children). Training in augmented and alternative communication, such as Intensive Interaction, was also considered. A range of programs was provided for children in early intervention and daycare sections, including center-based groups for four children, speech and language therapy, occupational therapy, and parent training sessions.

After two weeks of registration, all the admitted children had an individualized educational plan (IEP). During these two weeks, the evaluation was concluded in different sections by the trainer, SLP, OT, and the evaluation unit. Parents were members of the IEP session, and one day before the session, the family care staff needed to have a meeting session with them to make sure that they had decided on the aims and objectives for their child. The IEP was required to be updated every six months.

The dominant shortage in the care system for ASD is that the diagnosis and support system is poorly documented, mainly because support and services data are not systematically collected. Instead of being kept electronically, they are primarily paper-based and organized [35]. The aim was to collect non-aggregated data in a format suited for analysis or management. Therefore, daily training tasks were documented in three primary forms: play, exercise, and activities. By the end of 2021, 3000 approved and registered tasks were collected from the trainers and therapists in electronic format. The rehabilitation services were also documented as treatment plans (with determined aims and objectives and possibilities of following) in an electronic and monitorable format. By the end of 2021, more than 1000 approved and registered electronic treatment plans had been collected.

Each class had a full high-definition (HD) camera, and parents could follow their children via their mobile at home or in the center’s lobby through internal TV screens. A quality control system was established to check all the training sessions considering the fidelity forms for the applied training and intervention approaches. Comments about the sessions were sent to the trainers and therapist at the end of the sessions using the center’s internal automation system. The automation system is also used to keep the treatment plans, tasks, training sessions, and internal contacts between the different units of the center. Staff in the administration and services section (transportation, catering, chores) also had short courses on DDs and ASD and their features, and how to communicate with them.

The screening and evaluation department was established to document and register the clients in the electronic system of the center; for example, screening and assessment instruments validated in Iran were adopted for use in the organization, notably the Hiva screening scale and *Gilliam Autism Rating Scales, second edition (GARS2).* The third version of GARS was also applied, and data on parental impacts of caregiving were collected using an electronic data-saving system. Health-related and epidemiological information was collected. The dominant perspective was that “Poor data produce poor models: children with developmental disabilities deserve better” [34]. The electronic data collecting aim was to apply for the development of a suitable model for health and caregiving planning routinely and monitor the efficacy of the provided care. By the end of the first four months of 2022, collected data on the center’s development was presented in four international peer-reviewed journals on the applicability of GARS3 to the Kurdish population [36]. Comparison was made between GARS 2 and 3 [37], screening for ASD in Kurdistan Region [38] and impacts of elopement on parents (paper submitted for publication). It was imperative, because in KRI and Iraq, there is no formal and standardized structure for monitoring illness and analyzing health care needs [39]. This system was piloted in one center, and the ultimate aim was to set up the system in the entire area for all the daycare centers that provide ASD training and rehabilitation services. Using a network to routinely collect data on diagnoses of all referred individuals with ASD, a data system gathered epidemiological monitoring and health surveillance information. The system was able to manage healthcare data by collecting, storing, managing, and analyzing. Unfortunately, the development of the system was halted without prior notification. At the same time, all the considered aims and objectives mentioned in the approved action plan were met for the particular phase of the action plan. In a lack of personal, professional, and organizational supporting systems, the termination decision was taken by one authoritarian who financially supported the center.

## 4. Methodology

**Figure 1:**
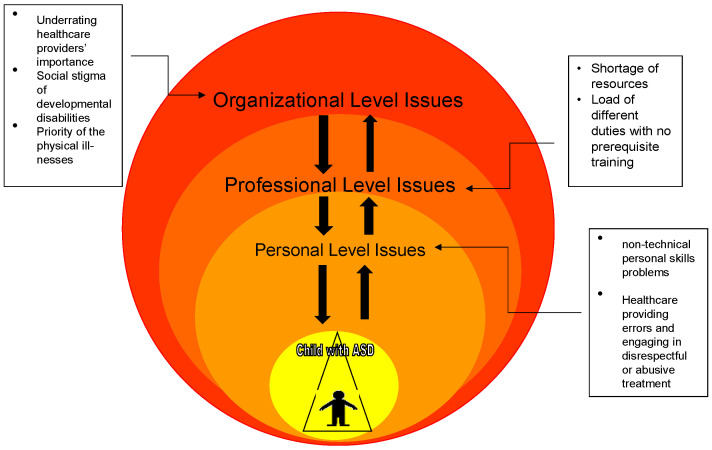
Impacts of the three considered organizational, professional, and personal factors in the healthcare provision system for individuals with ASD and their families in the KRI.

The basic concepts of systems theory were considered to understand the interplay between the three regarded factors of organizational, professional, and personal aspects of the healthcare system. The approach is used to understand a basic high-level integrated systems model as a framework to guide the investigation of the effect of these parts within the system that impact its effectiveness. Figure 1 depicts the model that is used in this review. Systems theory is the study of natural or human-made systems to understand their causal boundaries. The systems are influenced by their context, and are generally defined by their structure, idea, functioning, role, and relations with other systems in society. A system is a set of interrelated or interlinked units to form a unity or a cluster [40].

## 5. Discussion

The present report is prepared to answer why progress in healthcare service development has been deliberate in KRI for individuals with ASD, despite the news of promising activities in the region and several other countries. After two years of presence and coordinating work in the field, the reviewed barriers to developing healthcare systems in KRI were presented based on the ASD services establishment. Based on the present report’s considered aim, most short-term objectives were attained and possible to achieve. Hence, there are still severe barriers to facilitating an established system to continue its services based on the predetermined aims and approved long-term action plans. To finalize the present report and to present suggestions for the general healthcare services providers in the KRI region for individuals with ASD, and possibly other types of DDs and their caregivers, the following lessons were learned in the three personal, professional, and organizational levels in KRI:

### 5.1. At the Personal Level

The issue that might need highlighting at this level is countering the stigma of disability and its emotional impact on families and staff. However, the effects might be seen in different ways. For families, it might be the wish to have the child cured, and for staff, it might be reflected in a lack of motivation and its consequences in service provision. A better understanding of diversity in the region is needed, and to prove what works for particular minority and majority groups in support and intervention for the children with ASD, their parents, caregivers, and members of the extended family, immediate and broader communities, and environments. This understanding might help with impeding factors in both technical and non-technical skills

Considering the impact of adversity on healthcare and its developmental outcomes on different groups is also crucial. It also helps to understand the stigma and cultural clichés regarding ASD and other types of DDs, and the application of reliable and valid identification and outcomes protocols and scales that can be applied across settings within, and adjustable to, different group need in KRI and across the country. This will enable comparisons for preventive the degenerative developmental conditions, early home services, and clinical provision, suitable culturally service development, and sharing of expertise and resources that need to be considered. Currently, there is a lack of data in KRI, and healthcare services are provided with very short-term aims and objectives to resolve daily needs. Considering the diversity factors might increase the suitability and relevance of the official training and curriculum content to prepare healthcare providers at a personal level.

### 5.2. At the Professional Level

At this level, the emphasis could be on providing training to different groups of parents, but crucially to staff who may have little exposure to ASD. This is linked with the need to monitor the standard of care offered through record keeping and supervision. Sharing the healthcare services duties with non-medical professionals and recognizing non-medical doctors’ value in the caregiving services establishments such as healthcare management is another point that deserves to be considered. To recruit non-medical doctors to offer healthcare services provision independently through providing them with necessary training, mentorship, and professional development in different fields of ASD.

Focusing on common developmental disabilities and neurodevelopmental and neurodegenerative disorders, particularly ASD, learning disabilities (LD), cerebral palsy (CP), and attention deficit and hyperactivity disorder (ADHD). Diagnosis and awareness about developmental disabilities (ASD, ADHD, and other developmental delays) have increased in community and professional sectors, requiring more health and education services [41]. There is a need to get more information regarding the ‘potential routes to vulnerability to identify the vulnerable children in the region’. Items such as genetic, social, political, environmental, etc., or an amalgamation and interplay of different factors must be considered by different groups of medical and non-medical professionals.

### 5.3. At an Organizational Level

A primary public health services provider goal in LMICs is to improve healthcare quality. High-quality services result in increased service utilization, and improved outcomes heavily depend on the ability and knowledge of individuals who provide these services [5]. The focus here is on management and a desire for a cultural shift towards a more consultative and devolved management style, instead of the dominant authoritarian style of managing in which one person takes the entire responsibility based on their power. Likewise, the engagement with parents as partners, not as customers, is another tension.

Considering the mechanisms, such as giving different in-service training opportunities for enhancing clinicians’ skill sets to diagnose and work with individuals with developmental disabilities, such as individuals with ASD in different age groups, needed to be planned. Besides the clinical services, establishing approaches such as developing the home and community-based services to cater to each age group’s needs is crucial. It is essential with under five-years-old children with ASD who need to be covered under the early intervention programs [42]. These services are person-centered care delivered in the home and caregiving centers. A variety of health and human services are able to provide these services, which address the needs of individuals with functional limitations who need assistance with everyday activities, like getting dressed or bathing. Home and community services are often designed to enable people to stay in their homes rather than move to care facilities. Two public health and human services categories exist for this type of support. A combination of both types of services is also possible. The program’s sustainability, longevity, and impact are also crucial. It might be attained through the involvement of an accredited organization, governmental support, engagement of key policymakers, considering shared goals and concrete objectives, and identifying measurable, feasible action plans for different phases of the program.

Another organizational issue is the rate of turnover in healthcare provision sectors, and the act of replacing an employee with a new one because of the position left is measured as a percentage rate. Different rates of turnover have been reported for the staff working in healthcare services. An organization’s turnover costs include employment, training, and productivity loss costs. The minimum cost rate of turnover in the field of mental health, as Waldman et al. [43] suggested, “represented a loss of >5 percent of the total annual operating budget.” According to available data, healthcare organizations and the services industries suffer when the staff terminate their affiliation [44]. This rate is much higher in KRI based on the collected data (Samadi, under review study).

### 5.4. Lessons Learned from the Experience in KRI

Many lessons about barriers to establishing healthcare can be derived from this report. The first lesson is that a noticeable concern related to the long-term outcomes for vulnerable children and young people is understanding different types of available professional and nonprofessional potential services, and the standards for qualified services absent in the area. The cost of providing appropriate support and interventions and finding different sources for their attainment also need to be considered. The main hope is to develop an evidence-based practice that is feasible and appropriate for the region to find trustworthy funding sources. The governmental and private sector needs to consider the following points:

- Present services and support focus on a particular age group (age range of 5 to 10), and there should be programs for different needs at the various stages of development;
- Services are focused on particular groups of children with specific ethnical backgrounds. Children with ASD of different Yazidi, Christian, and other existing minorities are rarely seen in public or private healthcare centers. Most of them have their special centers established by an international organization in isolation and without having contact with the local centers. Therefore, it is recommended to identify the differences and similarities in the diverse needs of children and adolescents with ASD who are from different cultural and racial backgrounds in KRI;
- To develop appropriate protocols and validated recognition and output scales, rubrics, and instruments to assess healthcare provision impacts and other developmental outcomes for children and adolescents that can be used in research and clinical practice in KRI;
- To present and introduce examples of applicable models in addressing the impact of the adverse need of different racial and cultural groups, by adapting or developing, validating, or developing appropriate and measurable diagnosis, classification and intervention instruments and scales, and developing different approaches to provide optimum services and to strengthen or update the present acceptable services;
- It is recommended to reduce reliance on the parental and caregivers’ payment and understand the importance of considering different fundraising activities, social celebrity engagement, or establishing links with the international supporting organization to shift the focus on assisting the registered children and adolescents to afford the expenses of caregiving that inevitably reduces the quality of the services and increases the financial burdens of the parents and caregivers.

Midterm action plans from the governmental and private sectors engaged with healthcare service provision in KRI are needed to attain the abovementioned objectives. Supporting research on healthcare for children and adolescents has been identified as the cornerstone of the mentioned action plans. Significant progress is required to be made in identifying several linked research areas, enabling the stakeholders to progress systematically with the shared objectives.

As for the second lesson, some other fields must be considered and programmed for. There is generally a severe dearth of healthcare services and research in adults with ASD. There are no applications or evidence-based programs to rehabilitate the adult with ASD or consider the role of the family and different needed psychoeducational programs. There are no data available regarding lifespan and the issues within this age range. There is a need to improve outcomes, develop, evaluate, and implement healthcare pathways, and reduce morbidity and mortality, such as suicide or death, because of the associated comorbidities with ASD. Some small-scale studies have been undertaken worldwide and in neighboring countries regarding comorbid conditions with ASD and other DDs that need to be considered in the healthcare system. For example, half of the population of individuals with ASD experience different forms of anxiety [45] that need to be considered in the long-term plans of healthcare provision. However, a significant gap is developing a well-defined research program across the region to help clarify the role of the factors listed above and identify the most effective, scalable, and feasible plans to address existing issues.

The third lesson is to define and reconsider the rule of the private sector investment in healthcare service development. The funding of services is another organizational issue worthy of review and especially access to support for poorer families. Embracing the private sector and its financing seems beneficial; however, relying solely on them might be risky since most of them retain the right for themselves to stop support and services at any time.

On the other hand, there is a contradiction between the ultimate goal of the official mental health service providers and private investigators. Because services are purchased through parental and charity money, having more children in the caregiving centers indicates more cash. At the same time, facilitating transition out of the center after acquiring the educational and rehabilitation goals is the primary endeavor of the service providers. This is a big challenge since it is translated as a loss of money by private investigators.

Finally, in the absence of formal ethical consideration in healthcare services provision, research, favorable moral opinions, and appropriate guidelines such as research sponsorship are critical for the work undertaken in KRI. The issue with copyright, licensing, and application of the diagnostic scales for ASD also needs to be considered. The presented review is from one particular geographical area, and its generalization to other countries with a similar level of development needs to be considered with caution. This point is a limitation of the present review. Hence there are many lessons in this review that might be helpful for other LMICs aiming to develop their services for individuals with ASD.

## 6. Conclusions

This report highlights the importance of understanding the challenges associated with establishing healthcare services at three levels, personal, professional, and organizational, with the desire to control them. Based on a systems perspective, quality of healthcare is an emergent property of the entire healthcare system [46]; the improvement of healthcare for individuals with ASD is based on the contribution of the system components at all personal, professional, and organizational levels.

Different approaches were practiced to address the associated challenges while establishing healthcare services. Techniques such as pre- and in-service training for the healthcare service providers in KRI were one of the practiced experiences. This approach might be productive in most of the LMICs, where there is a lack of general training and ambiguity in the professional description for healthcare service providers with non-technical and technical skills at a personal level. A similar approach also seems to be influential with the issues associated with healthcare establishment at the professional and organizational levels. Further research needs to be considered to understand that similar shortcomings at the personal, professional, and organizational levels in establishing healthcare services are applied to other LMICs.

## Data Availability

Not applicable.

## References

[1] Edition F. (2013). Diagnostic and statistical manual of mental disorders. Am. Psychiatr. Assoc..

[2] Centers for Disease Control and Prevention (2022). CDC—National Center for Health Statistics—Homepage. https://www.cdc.gov/ncbddd/autism/facts.html.

[3] Faye M.L., McArthur J.W., Sachs J.D., Snow T. (2004). The challenges facing landlocked developing countries. J. Hum. Dev..

[4] Wong P.P. (2011). Small island developing states. Wiley Interdiscip. Rev. Clim. Chang..

[5] Saraceno B., van Ommeren M., Batniji R., Cohen A., Gureje O., Mahoney J., Sridhar D., Underhill C. (2007). Barriers to improvement of mental health services in low-income and middle-income countries. Lancet.

[6] Cohen A., Kleinman A., Saraceno B. (2007). World Mental Health Casebook: Social and Mental Health Programs in Low-Income Countries.

[7] Lewin S., Munabi-Babigumira S., Glenton C., Daniels K., Bosch-Capblanch X., van Wyk B.E., Odgaard-Jensen J., Johansen M., Aja G.N., Zwarenstein M. (2010). Lay health workers in primary and community health care for maternal and child health and the management of infectious diseases. Cochrane Database Syst. Rev..

[8] Bhutta Z.A., Lassi Z.S., Pariyo G., Huicho L. (2010). Global experience of community health workers for delivery of health related millennium development goals: A systematic review, country case studies, and recommendations for integration into national health systems. Glob. Health Workforce Alliance.

[9] Nicol E., Turawa E., Bonsu G. (2019). Pre-and in-service training of health care workers on immunization data management in LMICs: A scoping review. Hum. Resour. Health.

[10] Elsabbagh M., Divan G., Koh Y.J., Kim Y.S., Kauchali S., Marcín C., Montiel-Nava C., Patel V., Paula C.S., Wang C. (2012). Global prevalence of autism and other pervasive developmental disorders. Autism. Res..

[11] Autism Rates by Country 2022. https://worldpopulationreview.com/country-rankings/autism-rates-by-country.

[12] Center for Kurdish Progress (2020). Kurdistan Region on Autism Research and Awareness. https://kurdishprogress.com/2020/03/02/kurdistan-region-on-autism-research-and-awareness/.

[13] Black M.M., Walker S.P., Fernald L.C., Andersen C.T., DiGirolamo A.M., Lu C., McCoy D.C., Fink G., Shawar Y.R., Shiffman J. (2017). Advancing Early Childhood Development: From Science to Scale 1: Early childhood development coming of age: Science through the life course Early Childhood Development Series Steering Committee HHS Public Access. Lancet.

[14] Layne D.M., Nemeth L.S., Mueller M., Martin M. (2019). Negative behaviors among healthcare professionals: Relationship with patient safety culture. Healthcare.

[15] Emerson E., Savage A. (2017). Acute respiratory infection, diarrhoea and fever in young children at-risk of intellectual disability in 24 low-and middle-income countries. Public Health.

[16] Tumlinson K., Jaff D., Stilwell B., Onyango D.O., Leonard K.L. (2019). Reforming medical education admission and training in low-and middle-income countries: Who gets admitted and why it matters. Hum. Resour. Health..

[17] Cruess R.L., Cruess S.R. (2006). Teaching professionalism: General principles. Med. Teach..

[18] Jha V., Bekker H.L., Duffy S.R., Roberts T.E. (2007). A systematic review of studies assessing and facilitating attitudes towards professionalism in medicine. Med. Educ..

[19] Dwyer J., Paskavitz M., Vriesendorp S., Johnson S. (2006). An urgent call to professionalize leadership and management in health care worldwide. Manag. Sci. Health.

[20] Linnander E.L., Mantopoulos J.M., Allen N., Nembhard I.M., Bradley E.H. (2017). Professionalizing healthcare management: A descriptive case study. Int. J. Health Policy Manag..

[21] Bergström A., Skeen S., Duc D.M., Blandon E.Z., Estabrooks C., Gustavsson P., Hoa D.T., Källestål C., Målqvist M., Nga N.T. (2015). Health system context and implementation of evidence-based practices—Development and validation of the Context Assessment for Community Health (COACH) tool for low-and middle-income settings. Implement. Sci..

[22] Moran A.M., Coyle J., Pope R., Boxall D., Nancarrow S.A., Young J. (2014). Supervision, support and mentoring interventions for health practitioners in rural and remote contexts: An integrative review and thematic synthesis of the literature to identify mechanisms for successful outcomes. Hum. Resour. Health.

[23] Woltmann E.M., Whitley R., McHugo G.J., Brunette M., Torrey W.C., Coots L., Lynde D., Drake R.E. (2008). The role of staff turnover in the implementation of evidence-based practices in mental health care. Psychiatr. Serv..

[24] Rosenbaum P., Gorter J.W. (2012). The ‘F-words’ in childhood disability: I swear this is how we should think!. Child Care Health Dev..

[25] Hussein R.A., Mahmoud R.A., AL-Hamadi N.Q. (2017). A comparative study to evaluate Primary Health Care centers with family and non-family Medicine doctors in Basra. Int. J. Multidiscip. Curr. Res..

[26] Al Hilfi T.K., Lafta R., Burnham G. (2013). Health services in Iraq. Lancet.

[27] Khedir H.H. (2021). IDPs in the Kurdistan Region of Iraq (KRI): Intractable Return and Absence of Social Integration Policy. Int. Migr..

[28] Ibrahim H., Hassan C.Q. (2017). Post-traumatic stress disorder symptoms resulting from torture and other traumatic events among Syrian Kurdish refugees in Kurdistan Region, Iraq. Front. Psychol..

[29] IOM, UNFPA, KRSO (2018). Demographic Survey Kurdistan Region of Iraq. http://iomiraq.net/reports/demographic-survey-kurdistan-region-iraq.

[30] Rosenbaum P., Stewart D. (2004). The World Health Organization International Classification of Functioning, Disability, and Health: A model to guide clinical thinking, practice and research in the field of cerebral palsy. Semin. Pediatr. Neurol..

[31] Raghavendra P., Bornman J., Granlund M., Björck-Åkesson E. (2007). The World Health Organization’s International Classification of Functioning, Disability and Health: Implications for clinical and research practice in the field of augmentative and alternative communication. Augment. Altern. Commun..

[32] Samadi S.A., Samadi H., McConkey R. (2015). A conceptual model for empowering families in less affluent countries who have a child with autism. Autism Spectrum Disorder-Recent Advances.

[33] Tilahun D., Hanlon C., Fekadu A., Tekola B., Baheretibeb Y., Hoekstra R.A. (2016). Stigma, explanatory models and unmet needs of caregivers of children with developmental disorders in a low-income African country: A cross-sectional facility-based survey. BMC Health Serv. Res..

[34] Damiano D., Forssberg H. (2019). Poor data produce poor models: Children with developmental disabilities deserve better. Lancet Glob. Health.

[35] Moramarco S., Basa F.B., Alsilefanee H.H., Qadir S.A., Gialloreti L.E. (2020). Developing a public health monitoring system in a War-torn Region: A field report from Iraqi Kurdistan. Disaster Med. Public Health Prep..

[36] Samadi S.A., Noori H., Abdullah A., Ahmed L., Abdalla B., Biçak C.A., McConkey R. (2022). The Psychometric Properties of the Gilliam Autism Rating Scale (GARS-3) with Kurdish Samples of Children with Developmental Disabilities. Children.

[37] Samadi S.A., Biçak C.A., Noori H., Abdalla B., Abdullah A., Ahmed L. (2022). Autism Spectrum Disorder Diagnostic Criteria Changes and Impacts on the Diagnostic Scales-Utility of the 2nd and 3rd Versions of the Gilliam Autism Rating Scale (GARS). Brain Sci..

[38] Samadi S.A., McConkey R., Nuri H., Abdullah A., Ahmad L., Abdalla B., Biçak C.A. (2022). Screening Children for Autism Spectrum Disorders in Low-and Middle-Income Countries: Experiences from the Kurdistan Region of Iraq. Int. J. Environ. Res. Public Health.

[39] World Health Organization (WHO) (2006). Health Systems Profile—Iraq. Regional Health Systems Observatory. http://apps.who.int/medicinedocs/documents/s17295e/s17295e.pdf.

[40] Papathanasiou I., Sklavou M., Kourkouta L. (2013). Holistic nursing care: Theories and perspectives. Am. J. Nurs. Sci..

[41] Boyle C.A., Boulet S., Schieve L.A., Cohen R.A., Blumberg S.J., Yeargin-Allsopp M., Visser S., Kogan M.D. (2011). Trends in the prevalence of developmental disabilities in US children, 1997–2008. Pediatrics.

[42] Kohli-Lynch M., Tann C.J., Ellis M.E. (2019). Early intervention for children at high risk of developmental disability in low-and middle-income countries: A narrative review. Int. J. Environ. Res. Public Health..

[43] Waldman J.D., Arora S. (2004). Measuring retention rather than turnover: A different and complementary HR calculus. Hum. Resour. Planning..

[44] Johnson L. (1999). Cutting Costs by Managing Nurse Turnover. Balance.

[45] Samadi S.A., McConkey R., Rodgers J. (2020). Assessing anxiety in Iranian children with Autism Spectrum Disorder. Res. Autism Spectr. Disord..

[46] Leveson N.G. (2004). A systems-theoretic approach to safety in software-intensive systems. IEEE Trans. Dependable Secur. Comput..

